# Supplementary material for: A high-resolution mRNA expression time course of embryonic development in zebrafish
Source: eLife. 2017 Nov 16;6:e30860. doi: 10.7554/eLife.30860 (PMC5690287; doi:10.7554/eLife.30860)
Supplement: Supplementary file 6. [file elife-30860-supp6.zip › biolayout-clusters-files/Cluster146-genes.html]

Cluster146


# Cluster146: Genes

| Ensembl ID | Gene Name | Chr | Start | End | Biotype |
| --- | --- | --- | --- | --- | --- |
| ENSDARG00000055292 | atg9b | 24 | 33916353 | 33949692 | protein\_coding |
| ENSDARG00000053636 | cracr2b | 25 | 3718923 | 3742331 | protein\_coding |
| ENSDARG00000022599 | dnpep | 6 | 13766864 | 13790901 | protein\_coding |
| ENSDARG00000001968 | dock5 | 8 | 6607705 | 6782040 | protein\_coding |
| ENSDARG00000054786 | faah2b | 7 | 24236966 | 24249709 | protein\_coding |
| ENSDARG00000055493 | hic1 | 15 | 25745779 | 25755002 | protein\_coding |
| ENSDARG00000017126 | ilvbl | 18 | 44775375 | 44798437 | protein\_coding |
| ENSDARG00000077400 | rbm10 | 23 | 849526 | 891460 | protein\_coding |
| ENSDARG00000059902 | vps16 | 6 | 8871524 | 8895304 | protein\_coding |
